# Immunoenhancement of Edible Fungal Polysaccharides (Lentinan, Tremellan, and Pachymaran) on Cyclophosphamide-Induced Immunosuppression in Mouse Model

**DOI:** 10.1155/2017/9459156

**Published:** 2017-11-20

**Authors:** Qian Zhang, Renhuai Cong, Minghua Hu, Yanhong Zhu, Xiangliang Yang

**Affiliations:** ^1^College of Life Science and Technology, Huazhong University of Science and Technology, Wuhan, China; ^2^Joint Laboratory for the Research of Pharmaceutics, Huazhong University of Science and Technology and Infinitus, Wuhan, China

## Abstract

Fungal polysaccharides display a variety of important biological activities, including anti-inflammatory, antitumor, and immune-stimulating activities. The aim of present study was to investigate the immunomodulatory effect of fungal polysaccharides on cyclophosphamide-induced immunosuppression in mice. Mice were pretreated orally with lentinan, tremellan, pachymaran, or a mixture of the three, respectively. The results showed that pretreatments with polysaccharides significantly increased the thymus index in cyclophosphamide-induced immunosuppression mice. The level of the cytokine IL-10 in sera of cyclophosphamide-induced mice was decreased after pretreatments of polysaccharides. Flow cytometry results showed that pretreatments with polysaccharides enhanced the phagocytosis of peritoneal macrophages in mice. The increased levels of serum antibody IgG and IgM were observed in the groups pretreated with polysaccharides. Our work demonstrated that the treatment of polysaccharides elicited strong immune activity and a protective effect against cyclophosphamide-induced immunosuppression.

## 1. Introduction

In everyday life, working pressure, pollution, immunosuppressive agents, and so on are the causes of lower immunity and subhealth [1]. Improving immunity to prevent diseases has long been one of the primary concerns for many researchers. The use of immune modulators to enhance the host defense responses can be an effective way to increase resistance to disease [2, 3]. Over the years, fungal polysaccharides have been explored for their broad spectrum of biological activities with relatively low toxicity and adverse effects [2, 4–7]. Some polysaccharides have been known to improve the immunity by activating immune-related cells, or promoting antibody production [2, 8]. Therefore, polysaccharides have received extensive attention in the fields of therapeutics.

A number of polysaccharides have been known to improve the immune activities and prevent immunosuppression. Pidotimod and red ginseng acidic polysaccharide,* Ganoderma atrum* polysaccharide,* Cheonggukjang* polysaccharide, and* Sargassum fusiforme* polysaccharide were reported to prevent immunosuppression in cyclophosphamide-induced mice [9–12]. Lentinan and pachymaran have been found to have immunostimulatory activities and exert indirect inhibitory effect on cancer cells [3, 13, 14].* Tremella* polysaccharides were reported to attenuate sepsis through inhibiting abnormal CD4 + CD25 high regulatory T cells in mice [15].* Tremella* polysaccharides also showed the antioxidant activity [16, 17]. Therefore, lentinan, tremellan, and pachymaran have the immunomodulation ability. However, it is unclear if oral administration of tremellan and pachymaran could protect mice from immunosuppression. The role of lentinan in improving the immune activities was unknown in cyclophosphamide-treated mice.

Cyclophosphamide-induced myelosuppression, immunosuppression, and oxidative stress lead to significant morbidity and mortality [5, 18–22]. IL-10 and IFN-*γ* play important roles in cyclophosphamide-induced immunosuppression. IL-10 is a multifunctional negative regulation factor and inhibits the proliferation of effector T cells. IFN-*γ* has been reported to be a key molecule to trigger the activation of cytokines [23]. Cyclophosphamide is one of the most widely used alkylating agents for inducing immunosuppression. The model of cyclophosphamide-induced immunosuppression was employed to evaluate the effects of immunotherapy of immunomodulators (including polysaccharides) [24].

In this study, we investigated the effects of polysaccharides (lentinan, tremellan, pachymaran, or a mixture of the three, resp.) on immune suppression in the cyclophosphamide-induced mouse model. Interestingly, these fungal polysaccharides enhanced thymus index and spleen index, improved the phagocytosis of peritoneal macrophages, decreased the levels of IL-10, and increased IgG and IgM antibody production in the mouse sera.

## 2. Methods

### 2.1. Materials and Reagents

Enzyme linked immunosorbent assay (ELISA) kits for interleukin-10 (IL-10), interferon-gamma (IFN-*γ*), and immunoglobulins (IgA, IgG, and IgM) were obtained from eBioscience (San Diego, CA). Cyclophosphamide was obtained from Baxter Oncology GmbH. Other reagents were purchased from commercial suppliers without any treatments, unless otherwise noted.

### 2.2. Preparation of Polysaccharides

The polysaccharide extracts were prepared through water extraction and alcohol precipitation [25]. Briefly, raw materials were refluxed with distilled water, filtrated, and concentrated. Subsequently, ethanol was added to water extracts until the alcohol concentration is up to 80%. Then, the deposition was collected and the final polysaccharide extracts were obtained after drying up. A polysaccharide mixture was obtained according to the proportion (lentinan : tremellan : pachymaran = 7 : 2 : 1).

The polysaccharides were dissolved separately in physiological saline under ultrasound at the concentration of 20 mg/mL and stored frozen at −20°C.

### 2.3. Animals and Administration

Female Balb/c mice (6–8 weeks old) were obtained from the Beijing HFK Bioscience Co. Ltd. (HFK). The animal studies were approved by the Animal Experimentation Ethics Committee of College of Life Science and Technology, Huazhong University of Science and Technology. All procedures were carried out in strict compliance with the animal care guidelines of the Science and Technology Department of Hubei Province.

**Scheme 1:**
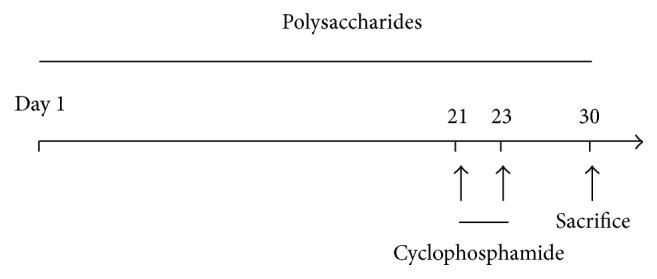
Scheme of animal experiment protocol. Mice were pretreated with oral polysaccharides or PBS for 30 days (from days 1 to 30). Cyclophosphamide (80 mg/kg) or PBS was injected intraperitoneally on days 21, 22, and 23. The mice were sacrificed on day 30.

The Balb/c mice were randomly divided into 6 groups—control group, model group, and polysaccharides groups (lentinan, tremellan, pachymaran, or mixture of three). The mice were given polysaccharides (200 mg/kg) for 30 days in the groups pretreated with polysaccharides, while the mice in the control and model groups were served with the same volume of PBS. From days 21 to 23, cyclophosphamide (80 mg/kg) was injected intraperitoneally to induce the immunosuppression in the model group and polysaccharides groups (Scheme 1). The mice of each group were weighed every day after treatment with cyclophosphamide. Sera, thymus, and spleens of mice were collected at the end of experiments.

### 2.4. Thymus Index and Spleen Index

The thymus and spleen were obtained sterilely and weighed immediately. The indexes were calculated according to the formula as follows: index (mg/g) = weight of thymus (spleen)/body weight.

### 2.5. Phagocytosis of Peritoneal Macrophages

At the end of experiment, peritoneal macrophages of mice were aseptically harvested through peritoneal lavage with 5 mL DMEM after intraperitoneal administration of 20% sheep erythrocyte suspension (200 *μ*L) on day 26. Macrophages were collected and seeded at a density of 2 × 10^5^ cells/well into the 6-well plate. Florescent microspheres (Invitrogen) were added at 1 × 10^7^/well. One hour later, cells were collected for flow cytometry analysis.

### 2.6. Analysis of Cytokine Production in the Sera

Serum samples were collected, aliquoted, and stored frozen at −20°C. Analysis of IL-10 and IFN-*γ* in serum samples was performed according to commercially ELISA kit manual.

### 2.7. Serum Antibody Analysis

Antibodies IgA, IgM, and IgG in sera were measured by ELISA according to the report [26]. Briefly, the diluted sera (1 : 100000 for IgG and IgA and 1 : 20000 for IgM) were added to the microtiter plates. The plates were washed with PBS-Tween followed by the addition of horseradish peroxidase- (HRP-) conjugated goat anti-mouse IgA/M/G. The enzyme reaction was stopped and absorbance value was detected through measuring the optical density (OD) at 450 nm.

### 2.8. Statistical Analysis

All statistical analyses were performed using the GraphPad Prism 5.0 graphing program (GraphPad Software, San Diego, CA, USA). Standard error of mean (SEM) was used in the results analysis. Significance level was set at a *P* value of 0.05.

## 3. Results

### 3.1. Protective Effects of Polysaccharides on Cyclophosphamide-Induced Immunosuppression

**Figure 1:**
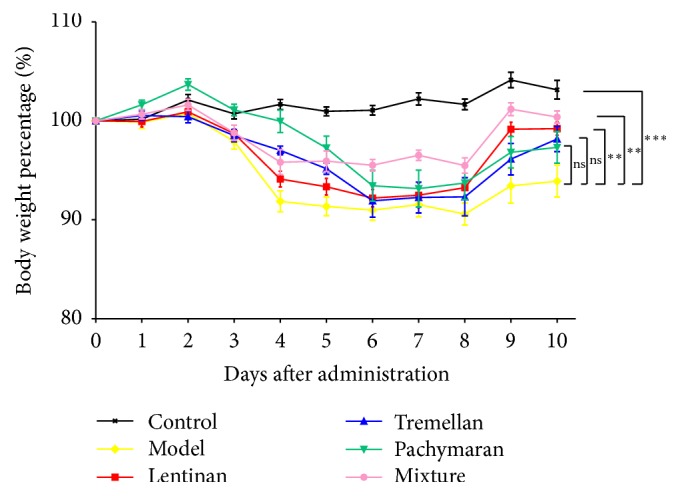
Polysaccharides pretreatment improved the protective effects from cyclophosphamide-induced immunosuppression. The change of mouse body weight after injection of cyclophosphamide (*n* = 9). The results represented means ± SEM. ^*∗∗*^*P* < 0.01, ^*∗∗∗*^*P* < 0.001, and ns: no significant difference.

Mouse bodyweight dropped largely in the model group (Figure 1), whereas the bodyweight increase was observed in the groups pretreated with polysaccharides. Compared to the model group, the mouse body weight recovered better, especially in the group pretreated with mixed polysaccharide.

**Figure 2:**
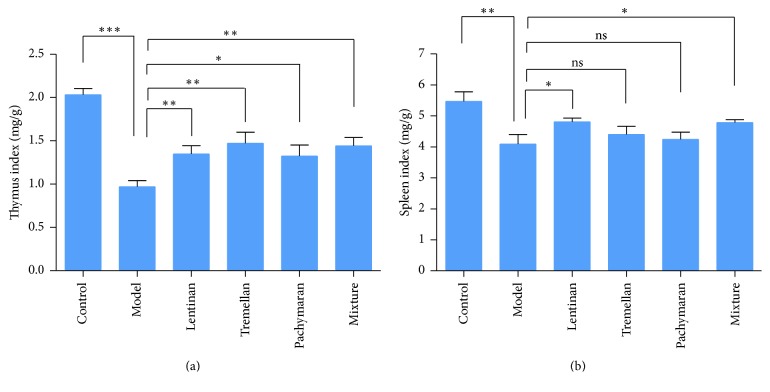
The changes of immune organ indexes after cyclophosphamide treatment (*n* = 9). (a) Thymus index of mice. (b) Spleen index of mice. The results represented means ± SEM. ^*∗*^*P* < 0.05, ^*∗∗*^*P* < 0.01, ^*∗∗∗*^*P* < 0.001, and ns: no significant difference.

Thymus index and spleen index in the model group were decreased significantly, whereas these indexes were increased after treatment with polysaccharides (Figure 2). Thymus index was increased significantly in all the groups pretreated with polysaccharides compared to that in the model group. Spleen index was increased significantly in the lentinan-treated and the mixture-treated groups compared to that in the model group.

### 3.2. The Increased Phagocytosis Capability of Macrophages

**Figure 3:**
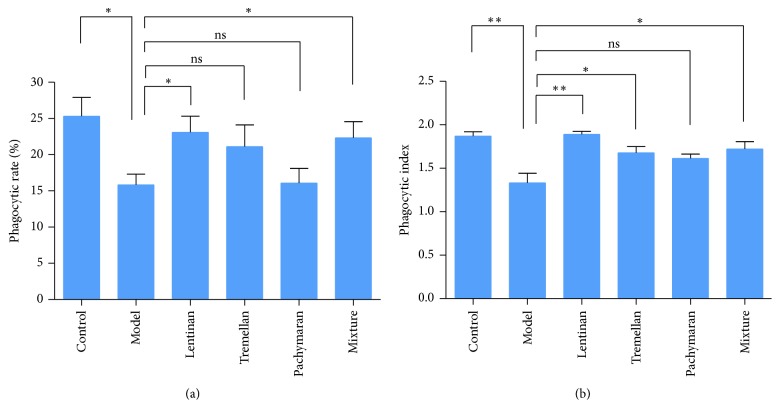
The phagocytosis of macrophages. (a) The phagocytic rate was determined by counting the number of macrophages phagocytosing florescent microspheres in a population of 100 macrophages. (b) The phagocytic index was determined by counting the number of phagocytosed florescent microspheres per 100 macrophages. The results represented means ± SEM (*n* = 4). ^*∗*^*P* < 0.05, ^*∗∗*^*P* < 0.01, and ns: no significant difference.

As shown in Figure 3, there was a marked inhibition of macrophage phagocytosis in the cyclophosphamide-treated mice compared to that in the control group (*P* < 0.05). However, treatment with oral polysaccharides augmented peritoneal macrophage phagocytosis, especially in the lentinan-treated and mixture-treated groups.

### 3.3. Cytokine Profiling after Treatment with Polysaccharides

**Figure 4:**
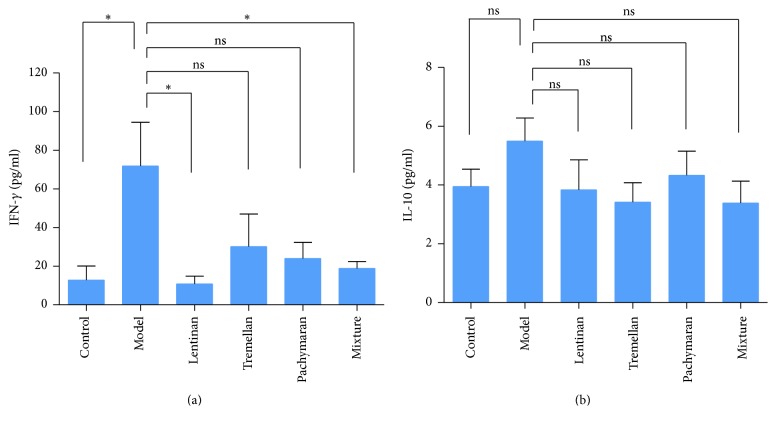
Cytokine production after polysaccharides pretreatment in the mouse model. The level of IFN-*γ* was decreased (a). The level of IL-10 showed no significant difference (b). The results represented means ± SEM (*n* = 4). ^*∗*^*P* < 0.05 and ns: no significant difference.

The level of cytokine IFN-*γ* was decreased significantly after treatment with polysaccharides compared to that in the model group (Figure 4(a)). The level of IL-10 in sera was higher in the model group, whereas it decreased after treatment with the polysaccharides, although there was no significant difference (Figure 4(b)). It indicated that immunosuppression can be improved partly after treatment with the polysaccharides.

### 3.4. Polysaccharides Elicited Antibody Response

**Figure 5:**
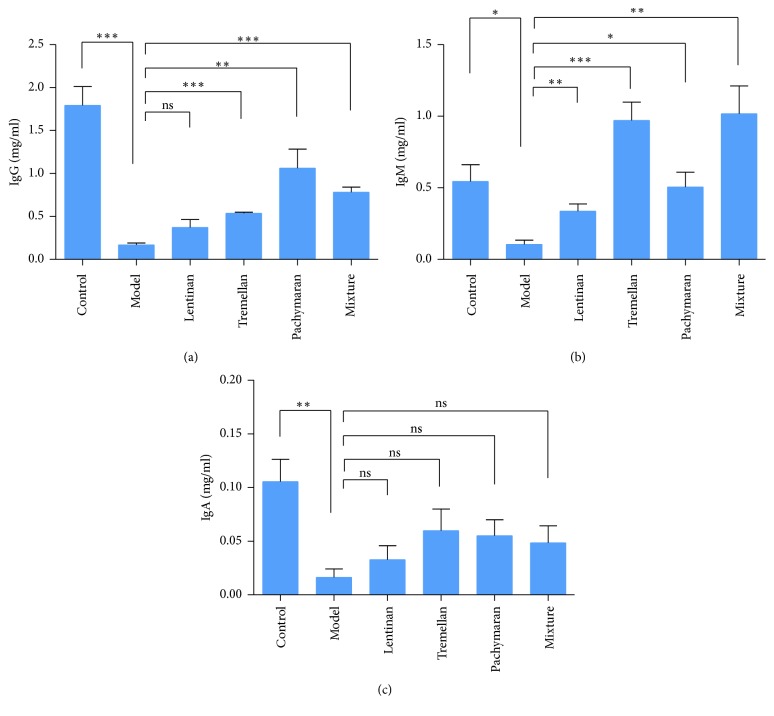
The production of IgG, IgM, and IgA in mouse sera after polysaccharides pretreatment. After treatment with polysaccharides, mouse sera were collected on day 30. (a, b) IgG and IgM production in sera were significantly increased after polysaccharides treatment. (c) IgA production in sera was increased, although there was no significant difference. The results represented means ± SEM (*n* = 4). ^*∗*^*P* < 0.05, ^*∗∗*^*P* < 0.01, ^*∗∗∗*^*P* < 0.001, and ns: no significant difference.

Serum samples were collected after treatments and the antibody production was quantified by ELISA. The levels of serum IgG and IgM in the polysaccharides pretreated groups were significantly increased compared to those in the model group (Figure 5). The levels of serum IgA in polysaccharide pretreated groups were also increased, although no significant difference was observed. Therefore, the treatments of polysaccharides enhanced the antibody response.

## 4. Discussion

The use of functional fungal polysaccharides from edible mushrooms for the development of biomedical drugs has attracted much attention. The numerous medical applications of fungal polysaccharides have been attributed to their antioxidative, antitumor, and immune-stimulating activities [27]. There are many reports that lentinan could increase immunity in immunosuppression patients, such as cancer patients [2]. However, few studies were conducted in unbiased way to directly investigate the role of tremellan and pachymaran in such protection. To address this issue, our study was focused on investigating the effect of three types of fungal polysaccharides on protection from immunosuppression.

As the important indicators of immune response, the thymus index and spleen index reflect the immune function of the organisms [28, 29]. The increased thymus index and spleen index were observed in the groups pretreated with fungal polysaccharides, suggesting that the oral polysaccharides could improve immunity in cyclophosphamide-treated mice. The recovered body weight in the groups pretreated with polysaccharides further demonstrated that the fungal polysaccharides could protect BALB/c mice from cyclophosphamide-induced immunosuppression.

The phagocytosis of macrophages is an initial response to pathogen microorganisms and one of the important indexes in evaluating innate immune response [12]. Our study showed that the increased phagocytosis of peritoneal macrophages was presented after polysaccharides pretreatment in the cyclophosphamide-treated mice, suggesting that the polysaccharides can enhance nonspecific immune response.

The IFN-*γ* is known as a critical proinflammatory cytokine. IFN-*γ* exerts strong regulatory influences on the proliferation, differentiation, and responses of B cell and T cell subsets [30]. In this study, the increased level of IFN-*γ* in the model group may be due to inflammatory response in experiments. The level of IFN-*γ* was decreased in the groups pretreated with polysaccharides compared to that in the model group. The increased level of anti-inflammatory cytokine IL-10 in the model group illustrated immunosuppression effect after the treatment of cyclophosphamide. But the level of IL-10 was decreased after treatment with different fungal polysaccharides. These results together demonstrated that fungal polysaccharides could improve the cyclophosphamide-induced immunosuppression, which is consistent with previous study [21].

IgA, IgG, and IgM are major immunoglobulins in humoral immune response. There are increasing evidences indicating that many kinds of polysaccharides can induce the production of IgA, IgM, and IgG [10, 31, 32]. Our results demonstrated that pretreatment of lentinan, tremellan, and pachymaran significantly enhanced the levels of IgG and IgM in immunosuppressed mice. The level of IgA was also increased, although there was no significant difference. These results demonstrated that fungal polysaccharides induced humoral immunity response through increasing production of antibody.

In summary, our findings indicated that orally administrated polysaccharides had strong protective effects in the cyclophosphamide-treated mice. Polysaccharides pretreatment enhanced thymus and spleen indexes, increased phagocytosis of macrophages, and induced humoral immunity response. Therefore, orally administrated fungal polysaccharide provided significant protection from immunosuppression in mice.
